# Brain CT can predict low lean mass in the elderly with cognitive impairment: a community-dwelling study

**DOI:** 10.1186/s12877-021-02626-8

**Published:** 2022-01-03

**Authors:** Yun-Ting Chen, Chiun-Chieh Yu, Yu-Ching Lin, Shan-Ho Chan, Yi-Yun Lin, Nai-Ching Chen, Wei-Che Lin

**Affiliations:** 1 Department of Diagnostic Radiology, Kaohsiung Chang Gung Memorial Hospital, Chang Gung University College of Medicine, No. 123 Ta-Pei Road, Niao-Sung Dist, Kaohsiung City, 83305 Taiwan; 2 Department of Medical Imaging and Intervention, Keelung Chang Gung Memorial Hospital, Chang Gung University College of Medicine, No. 222, Maijin Road, Anle Dist, Keelung City, 204201 Taiwan; 3 Department of Medical Imaging and Radiology, Shu-Zen Junior College of Medicine and Management, No. 452, Hwan-chio Road, Luju Dist, Kaohsiung City, 821004 Taiwan; 4 School of Nursing, Shu Zen College of Medicine and Management, No.452, Hwan-chio Road, Luju Dist, Kaohsiung, 821004 Taiwan; 5 Department of Neurology, Kaohsiung Chang Gung Memorial Hospital, Chang Gung University College of Medicine, No. 123, Ta-Pei Road, Niao-Sung Dist, Kaohsiung City, 83305 Taiwan

**Keywords:** Sarcopenia, Body composition, Cognitive impairement, Dementia, Brain atrophy, Computed tomography imaging

## Abstract

**Background:**

The coexistence of sarcopenia and dementia in aging populations is not uncommon, and they may share common risk factors and pathophysiological pathways. This study aimed to evaluate the relationship between brain atrophy and low lean mass in the elderly with impaired cognitive function.

**Methods:**

This cross-sectional study included 168 elderly patients who visited the multi-disciplinary dementia outpatient clinic at Kaohsiung Chang Gung Memorial Hospital for memory issues, between 2017 and 2019. The body composition was assessed by dual energy X-ray absorptiometry (DEXA) and CT based skeletal muscle index including L3 skeletal muscle index (L3SMI) and masseter muscle mass index (MSMI). The brain atrophy assessment was measured by CT based visual rating scale. Possible predictors of low lean mass in the elderly with cognitive impairement were identified by binary logistic regression. ROC curves were generated from binary logistic regression.

**Results:**

Among the 81 participants, 43 (53%) remained at a normal appendicular skeletal muscle index (ASMI), whereas 38 (47%) showed low ASMI. Compared with the normal ASMI group, subjects with low ASMI exhibited significantly lower BMI, L3SMI, and MSMI (all *p* < 0.05), and showed significant brain atrophy as assessed by visual rating scale (*p* < 0.001). The accuracy of predictive models for low ASMI in the elderly with cognitive impairment were 0.875, (Area under curve (AUC) = 0.926, 95% confidence interval [CI] 0.844–0.972) in model 1 (combination of BMI, GCA and L3SMI) and 0.885, (Area under curve (AUC) = 0.931, [CI] 0.857–0.979) in model 2 (combination of BMI, GCA and MSMI).

**Conclusions:**

Global cortical atrophy and body mass index combined with either L3 skeletal muscle index or masseter skeletal muscle index can predict low lean mass in the elderly with cognitive impairment.

**Supplementary Information:**

The online version contains supplementary material available at 10.1186/s12877-021-02626-8.

## Introduction

Dementia is characteristic of progressive cognitive decline involving one or more of the cognitive domains. As the population ages, the number of patients with dementia also increases, resulting in extensive burdens on personal health, familial relationships, economies, and societies in general [1]. There is growing evidence indicating a complex interrelationship between dementia and risk factors and certain non-communicable diseases, including physical inactivity, malnutrition, smoking, alcohol use, mild cognitive impairment, lack of anabolic hormones, persistent inflammatory reactions, diabetes, depression, and hypertension [2]. In aging populations, some of the known risks may present simultaneously, such as cognitive decline, physical inactivity, malnutrition, and sarcopenia, the amalgamation of which may accelerate the brain’s aging process [3, 4]. Clarifying the potential links and interactions by using available tools of measurement could help to prevent or alleviate adverse outcomes in aging/super-aged societies.

The prevalence of sarcopenia is significantly higher in individuals with cognitive impairment/dementia [5]. Furthermore, recent studies have identified it as a significant risk factor for cognitive deterioration [6]. Sarcopenia is associated with increased rates of functional impairment, disability, falls, and frailty, resulting in adverse health events [7, 8]. The coexistence of sarcopenia, cognitive impairment, and brain atrophy in aging populations may indicate shared common risk factors and pathophysiological pathways [8]. A recent study reported that physical frailty is associated with a longitudinal decline in global cognitive function in non-demented older adults, while cognitive impairment could accelerate physical impairment and negative outcomes in older persons [9]. Other studies suggest sarcopenia and low muscle mass are linked to cognitive impairment/dementia and brain atrophy [10–13]. However, evidence of an association between sarcopenia/low muscle mass and cognitive impairment/dementia among the elderly remains inconsistent and lacking [14]. Although several brain-muscle axes have been proposed, the precise mechanism remains unclear and controversial [15, 16]. Accumulating evidence indicates that non-pharmacological treatments such as optimized nutrition and physical activity can be valuable countermeasures to both sarcopenia/low muscle mass and dementia/mild cognitive impairment (MCI) in terms of treatment and prevention [17–22]. Therefore, understanding the relationship between sarcopenia and dementia, and the early detection of risk factors and signs, are essential for early intervention in order to maintain or decrease the rate of disease progression.

Currently, dual energy X-ray absorptiometry (DEXA) is considered the gold standard for the diagnosis of sarcopenia, and is recommended by the Asian Working Group for Sarcopenia (AWGS) 2019 [23, 24]. In addition, quantification of skeletal muscle and masseter muscle by using CT could effectively represent nutritional status and physical activity [25, 26]. Meanwhile, CT is also becoming a useful imaging tool for dementia assessment. Although MRI is more precise in the evaluation of brain volume, several reliable measurements using CT study including visual rating scales for assessing brain atrophy in aging and neurodegenerative diseases are now widely applied [27–32]. Several studies have also shown a significant correlation between whole-body muscle, lumbar skeletal muscle, and masseter muscle [33–38]. However, the association between sarcopenia and brain volume-related cognitive impairment remains unclear. Revealing the possible common risk factors, as well as potential relationships and pathophysiology may be helpful for the early detection and prevention of adverse events in aging societies.

In this study, we aimed to determine: 1) The possible risk factors and relationship between sarcopenia/low muscle mass and cognitive function impairment; 2) The association between body composition and brain atrophy; 3) To establish a model to accurately predict sarcopenia/low muscle mass in elderly Taiwanese with impaired cognitive function, which may be helpful for early risk detection to facilitate non-pharmacological intervention, improve quality of life, and reduce overall healthcare costs.

## Materials and methods

### Subjects

This cross-sectional study was conducted during the period of 2017 to 2019. All study participants visited the multi-disciplinary dementia outpatient clinic at Kaohsiung Chang Gung Memorial Hospital complaining of memory issues, accompanied by their family member(s). The demographic data and family history were recorded, and physical and neurological examinations were performed. All participants were evaluated by the consensus of a panel composed of neurologists, psychiatrists, and neuropsychologists to determine the presence or absence of dementia and its severity using the Clinical Dementia Rating (CDR). All participants consented to undergo cerebral and abdominal CT and dual-energy x-ray absorptiometry (DEXA). The study participants were selected according to the following criteria: age ≥ 65 years, CDR ≥ 0.5, no difficulty performing basic activities of daily living (ADL), no active treatment for cancer in the prior 3 years, and only cases with no hematoma, brain tumor, acute stroke, or post-infarction encephalomalacia resulting in mass effect or asymmetry affecting the ventricular system shape or subarachnoid space volume [39]. Patients with history of other neurologic or psychiatric illness, psychotropic medication usage, or substance abuse were excluded. Participants were divided into two groups (normal and low ASMI groups) according to the cut-off values (< 7.0 kg/m^2^ in men, and < 5.4 kg/m^2^ in women) for ASMI by AWGS 2019 reference. (See Supplemental Fig. 1) [23].

### Measurements of hand grip strength and gait speed

Hand grip strength (kg) was measured using a digital hand dynamometer. The grip strength of each hand was assessed twice; the maximal value of each hand was averaged as the final estimate of hand grip strength for analysis. The gait speed (m/s) was measured using a 6-m walking test at the usual pace.

### Head computed tomography (CT) data acquisition

All patients were in the supine position, with unenhanced CT scans performed of the vertex to base of skull using 256-row helical scanners with slice thickness of 5 mm, 120 kV, 225mAs. The scan angle was determined by the orbitomeatal line.

#### Visual rating of cerebral atrophy

Visual rating of clinical brain images was performed independently by three physicians, including a 2nd-year radiology resident (YTC) and two neuroimaging specialists (CCY and WCL), blind to all clinical information, using a 4-point global cortical atrophy (GCA) scale (See Supplement 1, Additional file 1 [40, 41]). A definite score would be discussed and assigned by three raters only when disputable results occurred.

#### Brain atrophy parameters of cerebral atrophy

**Fig. 1:**
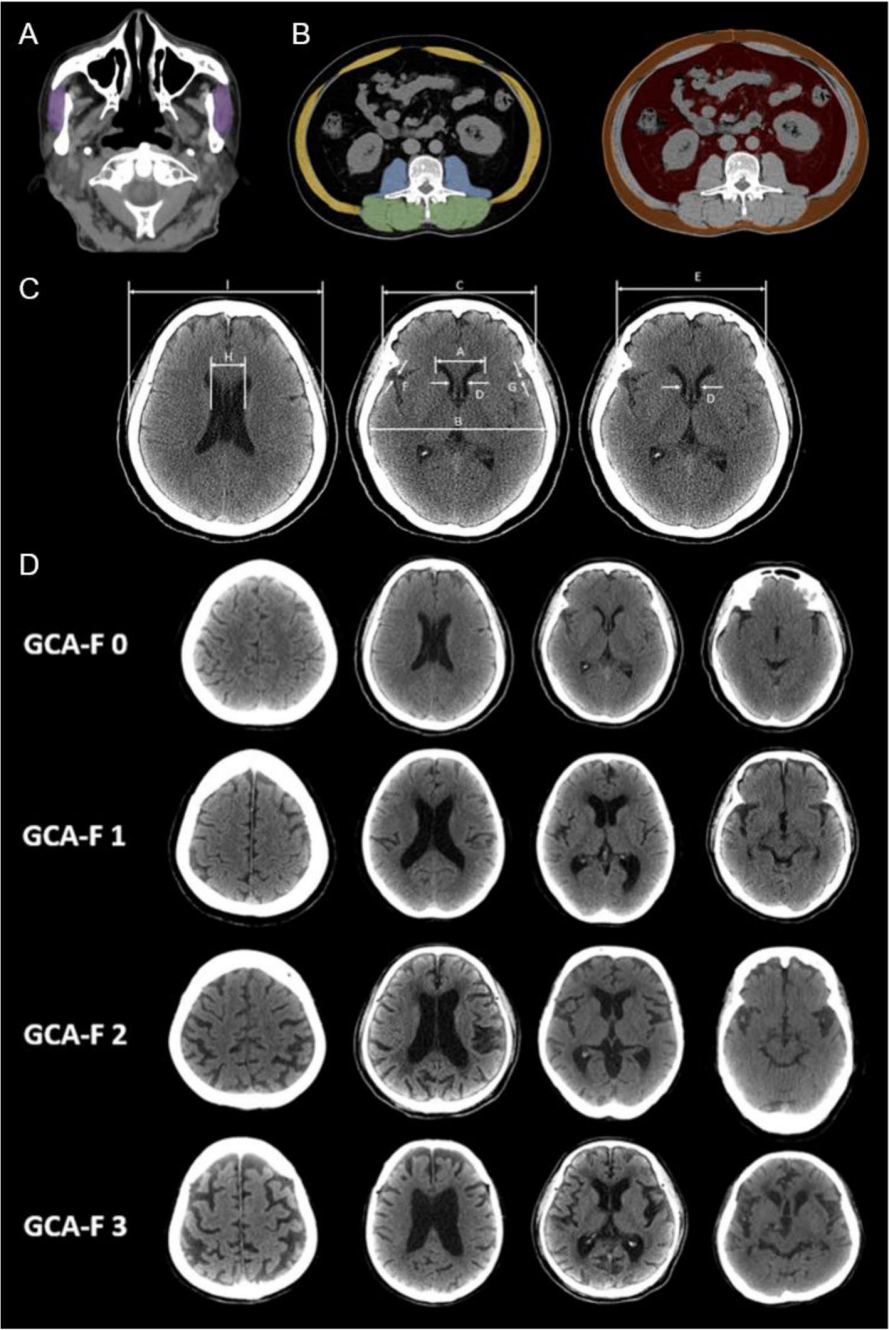
CT image analysis of body composition and brain atrophy. A. Head CT analysis of masseter muscle area. (Purple area: masseter muscle). B. Abdominal CT analysis of skeletal muscle area at third lumbar vertebral level. (Yellow area: abdominal wall muscle; Blue area: psoas muscle; Green area: paraspinal muscle; Orange area: subcutaneous adipose tissue; Red area: visceral adipose tissue). C. Measurements of brain atrophy parameters: A, B, C, D, E, F, G, H, I, in head CT images. Evans index (EI): ratio of maximum width of the frontal horns of the lateral ventricles (A) to the maximal internal diameter of the skull; Frontal horn index (FHI): ratio of the greatest external diameter of the frontal bone (C) to the greatest distance between the frontal horns at the same line (A); Bicaudate ratio (BCR): ratio of the maximum caudate nuclei distance (D) to distance between inner tables of skull at the same line (E); (B) at the same level; Sylvian fissure ratio (SFR): sum of bilateral width of the insular cisterns (in mm) (F)(G); Schiersmann index: ratio of the lateral ventricles greatest distance at the level of cella media (H) to distance between outer tables of skull (I) at the same line; Huckman number: sum of maximum width of the frontal horns of the lateral ventricles (in mm) (A) and the maximum caudate nuclei distance (in mm) (D). D. Examples of frontal cortical atrophy (F-GCA) scale. GCA 0: normal sulci and ventricle; GCA 1: slight widening of sulci with mild ventricular enlargement; GCA 2: gyral volume loss with moderate ventricular enlargement; GCA 3: pronounced widening of sulci with severe volume loss (knife blade atrophy) and severe ventricular enlargement

The brain atrophy parameters were measured on the CT scans, based on the commonly used method described by Meese [42], including Evans index (EI), Frontal horn index (FHI), Bicaudate ratio (BCR), Sylvian fissure ratio (SFR), Schiersmann index, and Huckman number (Fig. 1). Every parameter was measured twice, and the mean value was calculated to increase accuracy and limit the “partial volume” effect [39].

#### Head CT analysis of masseter cross-sectional area

Bilateral masseter cross-sectional area was quantified by evaluating axial measurements 2 cm below the zygomatic arch, as previously demonstrated in a study by Hu et al. Manual region of interest (ROI) was performed twice by primary author (YTC) under the supervision of a neuroradiologist with 3 years’ experience and the mean value was calculated. The average area of both masseter muscles was used for analysis (Fig. 1) [36, 43].

### Abdominal computed tomography (CT) data acquisition

All patients were in the supine position with unenhanced CT scans performed of the lower thorax to the pelvis using 256-row helical scanners, with slice thickness of 5 mm, 120 kV, 100–200 mAs.

#### Abdominal CT analysis of body composition

Three consecutive slices at the L3 vertebral body level were included, and measurements including area and attenuation of skeletal muscle and adipose tissue were averaged over these three images, as previously reported (See Supplement 2, Additional file 1 [44]) (Fig. 1). All measurements were taken by a 3rd-year radiology resident under the supervision of a musculoskeletal radiologist with 6 years’ experience [44].

### Dual energy x-ray absorptiometry (DEXA) examinations

Dual energy x-ray absorptiometry (DEXA) was used to estimate muscle mass. Subjects were clinically classified as low appendicular skeletal muscle mass (ASMI) according to appendicular skeletal muscle index (ASMI; ASM/height^2^) < 7.0 kg/m^2^ for men and < 5.4 kg/m^2^ for women, as recommended by the Asian Working Group for Sarcopenia (AWGS). In total, 38 low ASMI and 43 normal ASMI subjects participated in the study.

### Statistical analyses

Analyses were conducted using SPSS software (SPSS V.23, Chicago, IL, USA) and Stata software (Stata V.16.0). The demographic data, including age, sex, and CDR data were compared among the study groups using the 2-sample Student t-test, Pearson chi-square test, and Fisher’s exact test, and were reported as mean ± standard deviation (SD). The significance of differences in other demographic data, brain atrophy severity, and the body composition of the adipose tissue and muscle area were analyzed by analysis of covariance (ANCOVA) with the participant’s age and sex as covariates. Partial correlation analysis was performed with age and sex adjustments to determine associations among the body composition and brain atrophy visual rating scale with low ASMI. The threshold for statistical significance was set at *p* < 0.05. Factors associated with ASMI were analyzed using univariate logistic regression analysis, with the statistical significance set at *p* < 0.1. The significant factors in the univariate analysis were further analyzed using stepwise selection method with entry testing of binary logistic regression, adjusting for age and sex, to identify the predictors and calculate the odds ratios (OR) with 95% confidential intervals (95% CI). The ROC curves were constructed using the predictions (i.e., BMI, GCA, L3SMI, MSMI, model 1 (combination of BMI, GCA and L3SMI), and model 2 (combination of BMI, GCA and MSMI)) from the stepwise binary logistic regression model. Finally, Stata software was used to compare the two ROC curves with the statistical significance set at *p* < 0.05.

## Results

Participants were given an Institutional Review Board-approved study information sheet explaining the project objectives, and inclusion and exclusion criteria. Participants with age ≥ 65 years, and CDR ≥ 0.5 were included in our study. The exclusion criteria included age < 65 years, CDR = 0, having difficulty performing basic activities of daily living, under treatment for cancer in the prior 3 years, brain CT showed an organic brain lesion such as hematoma, brain tumor, acute stroke, or post-infarction encephalomalacia resulting in mass effect or asymmetry affecting the ventricular system shape or subarachnoid space volume, and missing data involving abdominal and brain CT, DEXA, demographic data and CDR [23]. Of 168 patients, 81 patients met the entry criteria to participate in the study and were divided into two groups (normal and low ASMI groups) according to the cut-off values for ASMI by AWGS 2019 reference. (See Supplemental Fig. 1).

### Demographic and clinical characteristics

The demographic and clinical data of the participants are shown in Table 1. The low ASMI and normal ASMI groups had similar mean age, gender distribution, and clinical dementia rating (CDR) scale level (age: *p* = 0.13; gender: *p* = 0.18; CDR: *p* = 1.00). There were no significant group differences in body height, grip strength, or gait speed (height: *p* = 0.10; grip strength: *p* = 0.73; gait speed: *p* = 0.78).

**Table 1:** Demographic characteristics

	Low ASMI (*n* = 38)^3^	Normal ASMI (*n* = 43)^3^	p^4^
Clinical demographic data^1^			
Age (years)	77.6 ± 4.8	75.6 ± 6.7	0.128
Gender (%)			
Male	17	13	0.177
Female	21	30	
Height (cm)	155.6 ± 8.7	152.2 ± 7.6	0.101
Body weight (kg)	54.1 ± 8.6	60.8 ± 10.6	< 0.001*
BMI (kg/m^2^)	22.3 ± 3.0	26.2 ± 4.0	< 0.001*
Grip strength (kg)	21.5 ± 8.9	20.6 ± 7.3	0.731
Gait speed (m/s)	0.9 ± 0.4	1.0 ± 0.3	0.777
CDR			
0.5	31	36	1.000
1	5	5	
2	2	2	
Assessments of brain atrophy^2^			
Visual rating scales			
GCA	26 ± 4	20 ± 4	< 0.001*
Brain atrophy index			
EI	0.35 ± 0.04	0.35 ± 0.04	0.439
FHI	0.28 ± 0.03	0.28 ± 0.03	0.247
BCR	0.16 ± 0.03	0.15 ± 0.03	0.525
SFR	0.06 ± 0.02	0.06 ± 0.02	0.734
Schiersmann index	0.29 ± 0.04	0.28 ± 0.04	0.652
Huckman index	57.50 ± 8.46	56.10 ± 8.20	0.884
Assessments of body composition^2^			
Dual energy x-ray absorptiometry			
ASMI (kg/m^2^)	5.54 ± 0.77	6.50 ± 0.83	< 0.001*
Cross-sectional CT image at the L3 vertebral body level			
Adipose tissue index (cm^2^/m^2^)			
Visceral adipose tissue	48.4 ± 28.5	63.0 ± 29.3	0.008*
Subcutaneous adipose tissue	29.4 ± 20.3	51.9 ± 36.8	0.001*
Skeletal muscle index (cm^2^/m^2^)			
Abdominal wall muscle	16.1 ± 3.3	19.2 ± 3.9	< 0.001*
Paraspinal muscle	14.8 ± 2.7	17.1 ± 2.9	< 0.001*
Psoas muscle	4.7 ± 1.6	5.3 ± 1.7	0.041*
Total muscle	35.5 ± 5.6	41.7 ± 6.6	< 0.001*
Assessments of body composition^2^			
Cross-sectional CT image at the L3 vertebral body level			
Attenuation (HU)			
Visceral adipose tissue	−82.4 ± 15.5	−88.9 ± 12.2	0.053
Subcutaneous adipose tissue	−108.9 ± 6.5	− 108.7 ± 5.5	0.794
Abdominal wall muscle	21.2 ± 9.7	21.3 ± 8.8	0.933
Paraspinal muscle	32.6 ± 6.6	30.5 ± 8.6	0.074
Psoas muscle	39.8 ± 4.3	41.0 ± 4.7	0.283
Total muscle	28.4 ± 7.2	27.7 ± 7.6	0.416
Cross- sectional CT image at 2 cm below the zygomatic arch			
Masseter muscle index	118.2 ± 25.4	152.75 ± 27.07	< 0.001*

Demographic data, cognitive function level, and assessments of brain atrophy and body composition in elderly patients with low ASMI and normal ASMI subjects

*Abbreviations*: *ASMI* Appendicular skeletal muscle index, *BCR* Bicaudate ratio, *BMI* Body Mass Index, *CDR* Clinical dementia rating scale, *EI* Evans index, *FHI* Frontal horn index, *GCA* Global cortical atrophy, *SFR* Sylvian fissure ratio

^1^Sex data were compared by Pearson chi-square test. CDR data were compared by Fisher’s exact test. Age data were compared by independent t test. The others demographic data were compared by analysis of covariance (ANCOVA) after controlling for age and sex

^2^Visual rating scales, brain atrophy index, and cross-sectional CT image data were compared by ANCOVA after controlling for age and sex

^3^Data are presented as mean ± standard deviation; ^4^* *p* < 0.05

### Assessments of brain atrophy

Table 1 demonstrates the significant differences between the two groups in terms of the Global cortical atrophy (GCA) scale (Low ASMI: 26 ± 4; Normal ASMI: 20 ± 4; *p* < 0.001), after controlling for sex and age. No statistically significant differences were found in other brain atrophy parameters, including the Evans index (*p* = 0.44), Frontal horn index (*p* = 0.25), Bicaudate ratio (*p* = 0.53), Sylvian fissure ratio (*p* = 0.73), Schiersmann index (*p* = 0.65), and Huckman index (*p* = 0.88) between the two groups.

### Assessments of body composition

The skeletal muscle index (SMI) and adipose tissue index (ATI) were compared between the two groups (Table 1). Significant differences between the two groups were identified in the SMI of the abdominal muscle (Low ASMI: 16.1 ± 3.3; Normal ASMI: Mean:19.2 ± 3.9; *p* < 0.001), paraspinal muscle (Low ASMI: 14.8 ± 2.7; Normal ASMI: 17.1 ± 2.9; *p* < 0.001), psoas muscle (Low ASMI: 4.7 ± 1.6; Normal ASMI: 5.3 ± 1.7; *p* = 0.041), total abdominal muscle (Low ASMI: 35.5 ± 5.6; Normal ASMI: 41.7 ± 6.6; *p* < 0.001), and masseter muscle (Low ASMI: 118.2 ± 25.4; Normal ASMI: 152.8 ± 27.1; *p* < 0.001), as well as the ATI of the visceral adipose tissue (VAT) (Low ASMI: 48.4 ± 28.5; Normal ASMI: 63.0 ± 29.3; *p* = 0.008) and subcutaneous adipose tissue (SAT) (Low ASMI: 29.4 ± 20.3; Normal ASMI: 51.9 ± 36.8; *p* = 0.001), after controlling for age and sex. There were no significant differences in attenuation of the skeletal muscle or adipose tissue between the two groups.

### Associations between ASMI and body composition parameter and visual rating scale

**Fig. 2:**
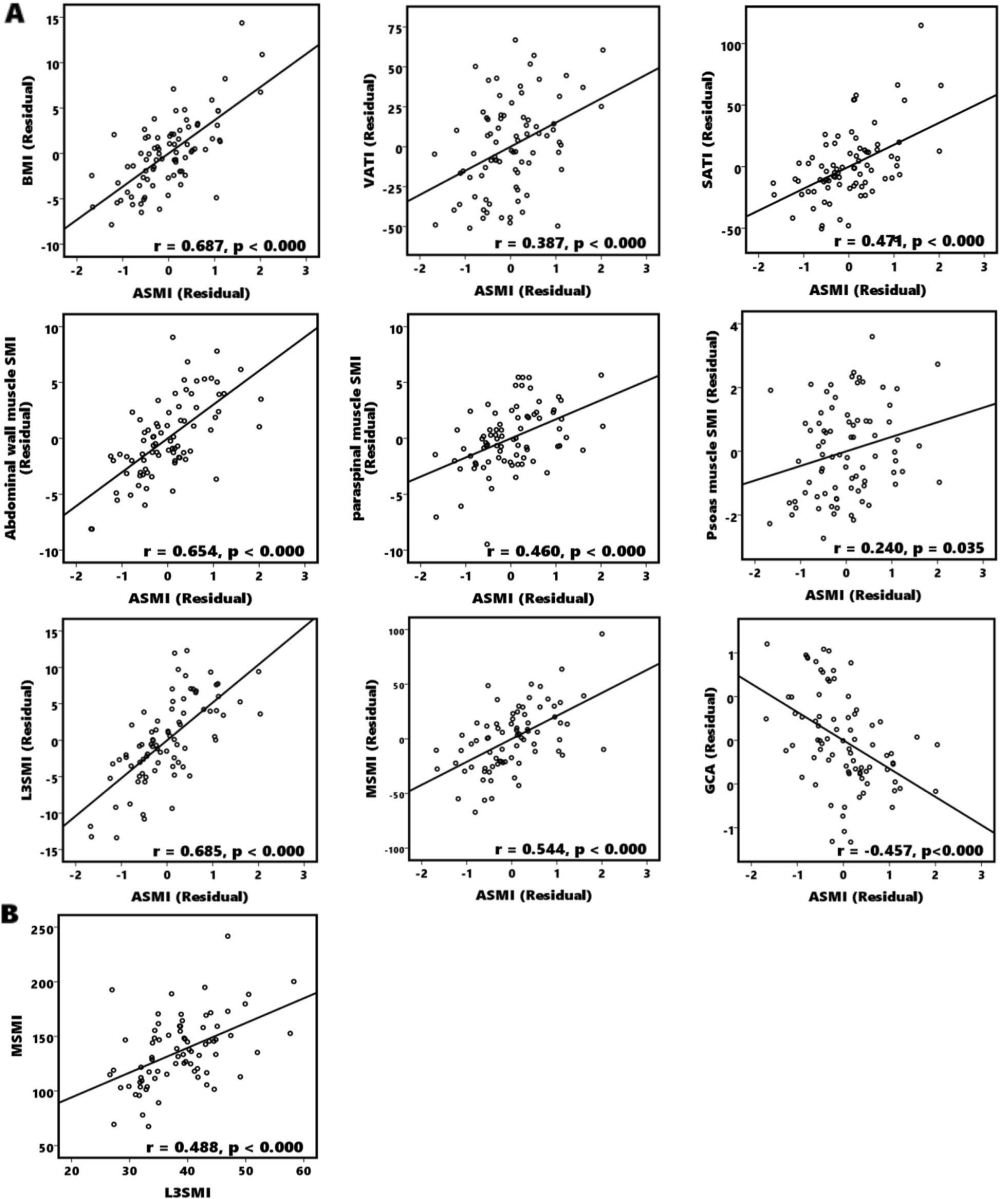
Associations between ASMI and body composition parameter and visual rating scale. The Partial correlation was conducted after controlling for age and sex variables. **A** A higher ASMI was positively correlated with higher BMI, L3 level ATI, L3 level SMI, and MSMI and negatively correlated with a higher GCA. **B** A higher L3SMI was positively correlated with higher MSMI. Abbreviations: ASMI, Appendicular skeletal muscle index; ATI, Adipose tissue index; BMI, Body mass index; GCA, Global cortical atrophy; L3SMI, L3 skeletal muscle index; MSMI, Masseter skeletal muscle index, SMI, Skeletal muscle mass index

Figure 2 outlines the correlations between ASMI and BMI, skeletal muscle index, adipose tissue index, and the GCA by using a partial correlation analysis controlling for age and sex. A higher ASMI was positively correlated with higher BMI (correlation confident(r) = 0.687, *p* < 0.001), visceral adipose tissue index (*r* = 0.387, *p* < 0.001), subcutaneous adipose tissue index (*r* = 0.471, *p* < 0.001), abdominal muscle index (*r* = 0.654, *p* < 0.001), paraspinal muscle index (*r* = 0.460, *p* < 0.001), psoas muscle index (*r* = 0.240, *p* < 0.035), total abdominal muscle index (*r* = 0.685, *p* < 0.001), and masseter muscle index (*r* = 0.551, *p* < 0.001). The higher ASMI was negatively correlated with a higher global cortical atrophy scale (*r* = − 0.457, *p* < 0.001). The correlation between L3SMI and MSMI was also investigated, revealing that a higher L3SMI was positively correlated with higher MSMI (*r* = 0.488, *p* < 0.001).

### Univariate analysis and logistic regression analysis

The results of the univariate analysis and multivariate analysis are presented in Table 2. A total of 6 variables (BMI, VATI, SATI, L3SMI, MSMI and GCA) showing a statistically significant odds ratio (*p* < 0.10) were entered into a stepwise binary regression model for the prediction of low ASMI. After adjusting for age and sex, BMI (*p* = 0.049, OR = 0.748), L3SMI (*p* = 0.003, OR = 0.705) and GCA (*p* = 0.002, OR = 1.50) were associated with a low ASMI. Considering the correlation between L3SMI and MSMI, we performed the binary logistic regression without the L3SMI variable, with the results revealing that BMI (*p* = 0.013, OR = 0.718), MSMI (*p* = 0.010, OR = 0.960) and GCA (*p* = 0.001, OR = 1.44) were associated with a low ASMI.

**Table 2:** Univariate analysis and multivariate analysis of factors associated with low lean mass

	Univariate Analysis		Multivariate Analysis^1^		Multivariate Analysis without L3SMI^1^	
Variable	OR (95% CI)	*p* value^2^	OR (95% CI)	*p* value^3^	OR (95% CI)	*p* value^3^
Age	1.062 (0.983, 1.147)	0.130				
Sex	1.868 (0.750, 4.652)	0.179				
BMI	0.702 (0.587, 0.841)	< 0.001*	0.748 (0.560, 0.999)	0.049*	0.718 (0.554, 0.932)	0.013*
VATI	0.983 (0.968, 0.999)	0.042*				
SATI	0.971 (0.952, 0.991)	0.004*				
L3SMI	0.843 (0.769, 0.924)	< 0.001*	0.705 (0.562, 0.885)	0.003*		
MSMI	0.946 (0.921, 0.971)	< 0.001*			0.960 (0.932, 0.990)	0.010*
GCA	1.399 (1.198, 1.633)	< 0.001*	1.496 (1.153, 1.941)	0.002*	1.442 (1.153, 1.802)	0.001*

*Abbreviations*: *ASMI* Appendicular skeletal muscle index, *BMI* Body mass index, *CI* Confidence interval, *GCA* Global cortical atrophy, *L3SMI* L3 skeletal muscle index, *MSMI* Masseter skeletal muscle index, *OR* Odds ratio, *SATI* Subcutaneous adipose tissue index, *SMI* Skeletal muscle index, *VATI* Visceral adipose tissue index

^1^The multivariate analysis was conducted after controlling for age and sex

^2^Univariate Analysis: * *p* < 0.1

^3^Multivariate Analysis: * *p* < 0.05

### Diagnostic value of BMI, GCA, and skeletal muscle index for predicting low lean mass

**Fig. 3:**
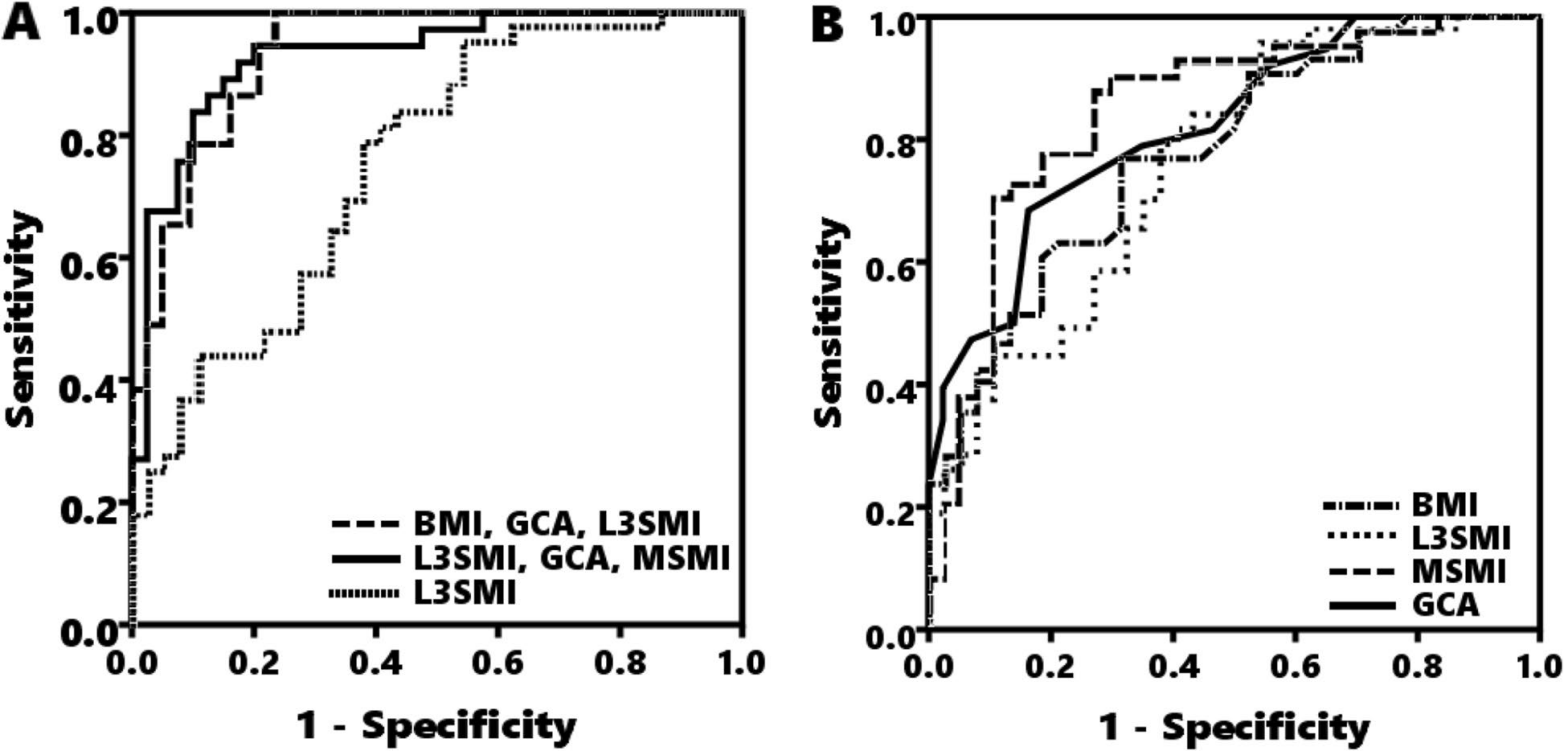
Receiver operating characteristic (ROC) curve for predicting low lean mass in cognitive impaired elderly. The ROC curves of BMI, GCA, L3SMI, MSMI, model 1 (combination of BMI, GCA, and L3SMI), and model 2 (combination of BMI, GCA, and MSMI) for predicting low lean mass. **A** The AUC of model 1 and model 2 was significantly greater than L3SMI. **B** There was no significant difference between BMI, GCA, L3SMI, and MSMI in the AUC. (Table 3) (See Supplemental Table 1, Additional file 2). Abbreviations: BMI, Body mass index; GCA, Global cortical atrophy; L3SMI, L3 skeletal muscle index; MSMI, Masseter skeletal muscle index

The ROC curves were constructed using the predictions from the logistic regression models. The sensitivity, specificity, positive predictive values, negative predictive values, positive likelihood ratio, negative likelihood ratio, standard error, and area under the curve of BMI, GCA, L3SMI, MSMI for model 1 (combination of BMI, GCA and L3SMI), and model 2 (combination of BMI, GCA and MSMI) are shown in Table 3. The AUC of model 1 (AUC: 0.926; accuracy: 0.875; [CI] 0.844–0.972) was significantly greater than BMI, GCA, and L3SMI individually (AUC: 0.777, 0.820, and 0.757; [CI] 0.672–0.863, 0.713–0.892, and 0.654–0.851; Bonferroni-adjusted *p*-value: 0.004, 0.020, and 0.002 respectively). The AUC of model 2 (AUC: 0.931; accuracy: 0.885; [CI] 0.857–0.979) was significantly greater than BMI, GCA, L3SMI, and MSMI individually (AUC: 0.777, 0.820, 0.757, and 0.827; [CI] 0.672–0.863, 0.713–0.892, 0.654–0.851, and 0.732–0.908; Bonferroni-adjusted p-value: 0.008, 0.048, 0.035, 0.014 respectively)(Table 3) (Fig. 3) (See Supplemental Table 1, Additional file 2). There was no significant difference between model 1 and model 2 in the AUC (Bonferroni-adjusted *p*-value = 1.000). The pairwise comparison of ROC curves are shown in Supplemental Table 1. There was no significant difference between BMI, GCA, L3SMI, and MSMI in the AUC. (See Supplemental Table 1, Additional file 2).

**Table 3:** Diagnostic value of BMI, GCA, and skeletal muscle index for predicting low lean mass

Sensitivity, Specificity, Positive and Negative Predictive Values and Receiver Operating Curve Model of the BMI, GCA, L3SMI, MSMI and combination of BMI, GCA and skeletal muscle index (either L3SMI or MSMI).									
Variable^1^	Sensitivity	Specificity	PPV	NPV	+LR	-LR	Accuracy	SE	AUC (95%CI)
BMI	0.790	0.628	0.722	0.733	2.121	0.335	0.704	0.051	0.777 (0.672, 0.863)
GCA	0.684	0.837	0.788	0.750	4.203	0.377	0.765	0.046	0.820 (0.713, 0.892)
L3SMI	0.622	0.791	0.719	0.708	2.970	0.479	0.713	0.054	0.757 (0.654, 0.851)
MSMI	0.865	0.707	0.844	0.778	2.955	0.191	0.782	0.049	0.827 (0.732, 0.908)
Model 1^a^	0.919	0.837	0.829	0.923	5.645	0.097	0.875	0.028	0.926 (0.844, 0.972)
Model 2^b^	0.919	0.830	0.853	0.917	6.279	0.095	0.885	0.029	0.931 (0.857,0.979)

*Abbreviations*: *AUC* Area under the curve, *BMI* Body mass index, *GCA* Global cortical atrophy, *L3SMI* L3 skeletal muscle index, *MSMI* Masseter skeletal muscle index, *NPV* Negative predictive values, *PPV* Positive predictive values, *SE* Standard error, *+LR* Positive likelihood ratio, *−LR* Negative likelihood ratio

^1^Significantly different with all variables (*p* < 0.05)

^a^Model 1: combination of BMI, GCA and L3SMI

^b^Model 2: combination of BMI, GCA and MSMI

## Discussion

Currently, brain CT is widely used in dementia workups as the first step brain diagnostic imaging method due to its faster acquisition speed, cost-effectiveness, availability, and ability to exclude other pathologies [27]. Additional information, such as masseter muscle mass and brain atrophy, can also be obtained by non-enhanced brain CT. Several reliable methods, including visual rating scale (GCA), brain atrophy parameters (EI, FHI, BCR, SFR, Schiersmann index, and Huckman number), voxel-based morphometry (VBM), and region of interest (ROI) analyses have been used in previous studies to assess brain atrophy/neurodegenerative disease. Although VBM and ROI analyses are often considered more accurate methods, they require prohibitively expensive techniques and specialized hardware. The global cortical atrophy (GCA) scale not only reflects cortical atrophy itself but also the degree of sulcal and ventricular dilatation secondary to atrophy of the white matter. Previous studies have demonstrated that the GCA scale can be evaluated using CT with substantial agreement, as compared to MRI [41, 45]. Due to its efficiency, accuracy, and ease to perform, we recommend using the GCA scale in radiology reports for early detection of the risk of lean mass loss to facilitate early intervention.

Clinically, some cognitively impaired elderly with low ASMI do not exhibit significant declines in physical performance, while others are unaware of their reduced physical activity. Nonetheless, early detection of undiagnosed sarcopenia/low muscle mass is important. Primary intervention, which includes resistance training and nutritional supplementation, is crucial. The DEXA evaluation is presently considered the gold standard technique both in research and in clinical practice for the assessment of muscle mass [46]. Several studies have shown a significant association between the CT-based cross-section muscle mass index, including MSMI and L3SMI, and the ASMI [35, 37]. .Its application for predicting morbidities in certain conditions (cirrhosis, pulmonary disease, severe traumatic brain injury, etc.) has also been reported, and is considered a marker of sarcopenia [47–50]. .However, its representative efficacy for determining overall body muscle mass remains debatable [51]. In our study, there was a significant correlation between both MSMI and L3SMI and the ASMI. The body mass index (BMI) has been used for evaluating nutritional status; as such, a lower BMI has been commonly recognized as a marker for poor nutrition status [52]. Of note, the association between late-life BMI and dementia is still inconclusive. Some studies have identified late-life obesity (or high BMI) as a protective state for dementia, commonly termed the “obesity paradox” [53–55]. In our study, a lower BMI is independently associated with lean body mass loss in cognitively impaired elderly, which may reflect poor nutrition and physical activity [56]. In addition, our study noted a positive correlation between brain volume and body muscle mass, as also demonstrated in a previous study [16]. The degree of brain atrophy in the low ASMI group was significantly higher than that of the normal ASMI group, suggesting that brain atrophy and muscle wasting may occur concurrently. One previous study has also revealed a strong relationship between lean mass and white matter volume in participants both with and without dementia [11].

Our logistic regression results showed that several body composition and brain atrophy parameters (BMI, L3SMI, MSMI, and GCA) were predictive of low ASMI independently of other possible confounders. However, none of these attained optimal cutoff values, with both sensitivity and specificity at > 80%, resulting in an AUC < 0.85. The combination of the BMI, GCA score, and one of SMI (L3SMI (model 1) or MSMI (model 2)) into a single risk score enhanced the predictive accuracy of low ASMI, with both sensitivity and specificity at > 80% and AUC > 0.9, and showed significantly better predictive accuracy than BMI, GCA, L3SMI, and MSMI individually. No statistically significant difference was found between the two models in predicting low ASMI, indicating that MSMI could serve as an alternative marker for body muscle mass loss.

Recent studies have also demonstrated that masseter muscle mass is a simple and effective tool for assessing sarcopenia in elderly populations [34–36, 50]. Additionally, it is regarded as an effective predictor of early mortality following traumatic brain injury in the elderly [34, 36, 50]. For this reason, we suggest using MSMI as part of a muscle wasting assessment as it provides equally valid results as L3SMI, and is more accessible and easier to perform due to the lack of specialized software or arduous image analysis. Furthermore, a clinician can concurrently obtain reliable information regarding brain atrophy and skeletal muscle loss in a simple, non-enhanced brain CT scan.

Previous studies have suggested that the mechanism of the brain-muscle axis remains unclear, and is considered to be multifactorial [15, 16]. In the present study, brain volume is positively correlated with body lean mass. Masseter muscle mass loss may limit the action of mastication, leading to poor nutrient intake and malnutrition. Malnutrition may further aggravate the loss of muscle mass, resulting in poor muscle strength and reduced physical activity. Abdominal muscle wasting may reflect a deficiency in core stability, low physical activity, and unsteadiness which collectively increase the risk of adverse events, including falls. In addition, muscle wasting may further affect brain volume by impairing certain physiological functions, such as glucose regulation, hormone production, and cellular communication, which can also regulate neuroinflammatory responses and lead to neuroendocrine dysregulation and brain atrophy [57, 58]. Recent evidence shows that adequate physical activity not only enhances muscle mass and strength, but also benefits cognitive function and brain volume in the elderly with cognitive deficits [59–62]. Several pathophysiology pathways of neurologic insults, affecting brain atrophy and muscle wasting simultaneously, have been demonstrated in previous studies, such as primary reward system deficit, visuomotor control failure, and gait and balance associated white matter alterations [63–67]. Furthermore, any condition that causes inflammation not only increases muscle catabolism and impairs myogenesis leading to muscle wasting, but also alters blood-brain barrier functions and reduces neural plasticity, resulting in neurodegenerative diseases and brain atrophy [68–71]. Chronic illnesses and other conditions leading to prolonged inactivity, inadequate nutrient intake, and poor nutrient absorption, may also result in both muscle wasting and neurodegeneration [72]. As such, early detection and intervention for elderly patients with a higher risk of brain atrophy and muscle wasting is necessary.

Several limitations to our study should indeed be mentioned. First, the population of our study consisted only of cognitively impaired elderly recruited from a single medical center, composing a relatively small sample size, thus limiting the generalizability of the results. Second, the study cannot infer causality between brain volume and body composition as the temporal sequence cannot be established in a cross-sectional study. To achieve this, further long-term longitudinal study is necessary. Third, measurement of masseter cross-sectional area was performed twice by a trained investigator manually to reduce the partial volume effect; however, the possibility of intra-examiner errors cannot be completely excluded. Several studies have documented automatic or semi-automatic segmentation methods in masseter muscle area measurement, while manual segmentation, offers more convenience and availability for clinical physicians. In addition, future research should involve a multi-center prospective analysis, and include additional patient characteristics, such as dental status, nutrition status, and inflammatory biomarkers which may affect both muscle mass and brain atrophy, and provide more conclusive evidence regarding brain atrophy. Finally, further research is needed to provide evidence of applicability in other population groups, such as the elderly with normal cognitive function, the elderly with known neurodegenerative diseases, and cerebral atrophy in younger persons.

In conclusion, we herein demonstrate a significant association between low lean mass and brain volume loss in elderly Taiwanese with cognitive deficiency. The combination of BMI, GCA, and SMI, either L3SMI or MSMI, achieved good predictive accuracy for low ASMI. Early detection of such patients is necessary to facilitate further treatment and prevent adverse events, thereby improving quality of life in the elderly. Further clarification of the underlying mechanisms of muscle wasting, and future studies involving additional parameters and various conditions are required.

## Supplementary Information

**Additional file 1 **: **Supplement 1**. Visual rating of global cortical atrophy. **Supplement 2**. Abdominal CT analysis of body composition at L3 level.

**Additional file 2 **: **Supplemental Table 1.** Pairwise comparison of ROC curves between BMI, GCA, L3SMI, MSMI, Model1 and Model 2.

**Additional file 3 **: **Supplemental Figure 1**. Participant flow charts.

## Data Availability

The datasets generated and/or analysed during the current study are not publicly available due to patient privacy and Institutional Review Boards (IRB) disapproval, but are available from the corresponding author on reasonable request.

## Abbreviations

ADL: Activities of daily living
ANCOVA: Analysis of covariance
ASMI: Appendicular skeletal muscle index
ATI: Adipose tissue index
AUC: Area under the Curve
AWGS: Asian Working Group for Sarcopenia
BCR: Bicaudate ratio
BMI: Body mass index
CCY: Chiun Chieh Yu
CDR: Clinical Dementia Rating
CI: Confidential intervals
CRP: C-Reactive protein
CT: Computed tomography
DEXA: Dual energy X-ray absorptiometry
EI: Evans index
FHI: Frontal horn index
GCA: Global cortical atrophy
L3SMI: L3 skeletal muscle index
MCI: Mild cognitive impairment
MRI: Magnetic resonance imaging
MSMI: Masseter muscle mass index
OR: Odds ratios
ROC: Receiver operating characteristic
ROI: Region of interest
SAT: Subcutaneous adipose tissue
SD: Standard deviation
SFR: Sylvian fissure ratio
SPSS: Statistical Product and Service Solutions
SMI: Skeletal muscle mass index
VAT: Visceral adipose tissue
VBM: Voxel-based morphometry
WCL: Wei-Che Lin
YTC: Yun Ting Chen

## References

[1] Livingston G, Huntley J, Sommerlad A, Ames D, Ballard C, Banerjee S, Brayne C, Burns A, Cohen-Mansfield J, Cooper C (2020). Dementia prevention, intervention, and care: 2020 report of the lancet commission. Lancet.

[2] Organization WH (2017). Global action plan on the public health response to dementia 2017–2025.

[3] Huang CY, Hwang AC, Liu LK, Lee WJ, Chen LY, Peng LN, Lin MH, Chen LK (2016). Association of dynapenia, sarcopenia, and cognitive impairment among community-dwelling older Taiwanese. Rejuvenation Res.

[4] Robertson DA, Savva GM, Kenny RA (2013). Frailty and cognitive impairment--a review of the evidence and causal mechanisms. Ageing Res Rev.

[5] Pacifico J, Geerlings MAJ, Reijnierse EM, Phassouliotis C, Lim WK, Maier AB (2020). Prevalence of sarcopenia as a comorbid disease: a systematic review and meta-analysis. Exp Gerontol.

[6] Nishiguchi S, Yamada M, Shirooka H, Nozaki Y, Fukutani N, Tashiro Y, Hirata H, Yamaguchi M, Tasaka S, Matsushita T (2016). Sarcopenia as a risk factor for cognitive deterioration in community-dwelling older adults: a 1-year prospective study. J Am Med Dir Assoc.

[7] Janssen I (2006). Influence of sarcopenia on the development of physical disability: the cardiovascular health study. J Am Geriatr Soc.

[8] Kim M, Won CW (2019). Sarcopenia is associated with cognitive impairment mainly due to slow gait speed: results from the Korean frailty and aging cohort study (KFACS). Int J Environ Res Public Health.

[9] Lauretani F, Maggio M, Ticinesi A, Tana C, Prati B, Gionti L, Nouvenne A, Meschi T (2018). Muscle weakness, cognitive impairment and their interaction on altered balance in elderly outpatients: results from the TRIP observational study. Clin Interv Aging.

[10] Atkinson HH, Cesari M, Kritchevsky SB, Penninx BW, Fried LP, Guralnik JM, Williamson JD (2005). Predictors of combined cognitive and physical decline. J Am Geriatr Soc.

[11] Burns JM, Johnson DK, Watts A, Swerdlow RH, Brooks WM (2010). Reduced lean mass in early Alzheimer disease and its association with brain atrophy. Arch Neurol.

[12] Nourhashémi F, Andrieu S, Gillette-Guyonnet S, Reynish E, Albarède JL, Grandjean H, Vellas B (2002). Is there a relationship between fat-free soft tissue mass and low cognitive function? Results from a study of 7,105 women. J Am Geriatr Soc.

[13] Ohta Y, Nomura E, Hatanaka N, Osakada Y, Matsumoto N, Sasaki R, Tsunoda K, Takemoto M, Tadokoro K, Hishikawa N (2019). Female dominant association of sarcopenia and physical frailty in mild cognitive impairment and Alzheimer's disease. J Clin Neurosci.

[14] Noh H-M, Oh S, Song HJ, Lee EY, Jeong J-Y, Ryu O-H, Hong K-S, Kim D-H (2017). Relationships between cognitive function and body composition among community-dwelling older adults: a cross-sectional study. BMC Geriatr.

[15] Moon Y, Choi YJ, Kim JO, Han SH (2018). Muscle profile and cognition in patients with Alzheimer's disease dementia. Neurol Sci.

[16] Kilgour AH, Todd OM, Starr JM (2014). A systematic review of the evidence that brain structure is related to muscle structure and their relationship to brain and muscle function in humans over the lifecourse. BMC Geriatr.

[17] Pillard F, Laoudj-Chenivesse D, Carnac G, Mercier J, Rami J, Rivière D, Rolland Y (2011). Physical activity and sarcopenia. Clin Geriatr Med.

[18] Robinson S, Cooper C, Aihie Sayer A (2012). Nutrition and sarcopenia: a review of the evidence and implications for preventive strategies. J Aging Res.

[19] Morris MC (2016). Nutrition and risk of dementia: overview and methodological issues. Ann N Y Acad Sci.

[20] Vlachos GS, Scarmeas N (2019). Dietary interventions in mild cognitive impairment and dementia. Dialogues Clin Neurosci.

[21] Kouloutbani K, Karteroliotis K, Politis A (2019). The effect of physical activity on dementia. Psychiatriki.

[22] Tabei KI, Satoh M, Ogawa JI, Tokita T, Nakaguchi N, Nakao K, Kida H, Tomimoto H (2018). Cognitive function and brain atrophy predict non-pharmacological efficacy in dementia: the Mihama-Kiho scan Project2. Front Aging Neurosci.

[23] Chen LK, Woo J, Assantachai P, Auyeung TW, Chou MY, Iijima K, Jang HC, Kang L, Kim M, Kim S (2020). Asian Working Group for Sarcopenia: 2019 consensus update on sarcopenia diagnosis and treatment. J Am Med Dir Assoc.

[24] Buckinx F, Landi F, Cesari M, Fielding RA, Visser M, Engelke K, Maggi S, Dennison E, Al-Daghri NM, Allepaerts S (2018). Pitfalls in the measurement of muscle mass: a need for a reference standard. J Cachexia Sarcopenia Muscle.

[25] Yeh DD, Ortiz-Reyes LA, Quraishi SA, Chokengarmwong N, Avery L, Kaafarani HMA, Lee J, Fagenholz P, Chang Y, DeMoya M, Velmahos G (2018). Early nutritional inadequacy is associated with psoas muscle deterioration and worse clinical outcomes in critically ill surgical patients. J Crit Care.

[26] Hwang Y, Lee YH, Cho DH, Kim M, Lee DS, Cho HJ (2020). Applicability of the masseter muscle as a nutritional biomarker. Medicine (Baltimore).

[27] Adduru V, Baum SA, Zhang C, Helguera M, Zand R, Lichtenstein M, Griessenauer CJ, Michael AM (2020). A method to estimate brain volume from head CT images and application to detect brain atrophy in Alzheimer disease. Am J Neuroradiol.

[28] Stephen R, Liu Y, Ngandu T, Antikainen R, Hulkkonen J, Koikkalainen J, Kemppainen N, Lötjönen J, Levälahti E, Parkkola R (2019). Brain volumes and cortical thickness on MRI in the Finnish geriatric intervention study to prevent cognitive impairment and disability (FINGER). Alzheimers Res Ther.

[29] Khoury R, Ghossoub E (2019). Diagnostic biomarkers of Alzheimer’s disease: a state-of-the-art review. Biomark Neuropsych.

[30] Young PNE, Estarellas M, Coomans E, Srikrishna M, Beaumont H, Maass A, Venkataraman AV, Lissaman R, Jiménez D, Betts MJ (2020). Imaging biomarkers in neurodegeneration: current and future practices. Alzheimers Res Ther.

[31] Lin Y, Fu Y, Zeng YF, Hu JP, Lin XZ, Cai NQ, Weng Q, Zhao YJ, Lin Y, Cao DR, Wang N (2020). Six visual rating scales as a biomarker for monitoring atrophied brain volume in Parkinson's disease. Aging Dis.

[32] Aljondi R, Szoeke C, Steward C, Yates P, Desmond P (2019). A decade of changes in brain volume and cognition. Brain imaging Behav.

[33] Swartz JE, Pothen AJ, Wegner I, Smid EJ, Swart KM, de Bree R, Leenen LP, Grolman W (2016). Feasibility of using head and neck CT imaging to assess skeletal muscle mass in head and neck cancer patients. Oral Oncol.

[34] Tanabe C, Reed MJ, Pham TN, Penn K, Bentov I, Kaplan SJ (2019). Association of Brain Atrophy and Masseter Sarcopenia with 1-year mortality in older trauma patients. JAMA Surg.

[35] Umeki K, Watanabe Y, Hirano H, Edahiro A, Ohara Y, Yoshida H, Obuchi S, Kawai H, Murakami M, Takagi D (2018). The relationship between masseter muscle thickness and appendicular skeletal muscle mass in Japanese community-dwelling elders: a cross-sectional study. Arch Gerontol Geriatr.

[36] Wallace JD, Calvo RY, Lewis PR, Brill JB, Shackford SR, Sise MJ, Sise CB, Bansal V (2017). Sarcopenia as a predictor of mortality in elderly blunt trauma patients: comparing the masseter to the psoas using computed tomography. J Trauma Acute Care Surg.

[37] Portal D, Hofstetter L, Eshed I, Dan-Lantsman C, Sella T, Urban D, Onn A, Bar J, Segal G (2019). L3 skeletal muscle index (L3SMI) is a surrogate marker of sarcopenia and frailty in non-small cell lung cancer patients. Cancer Manag Res.

[38] Shen W, Punyanitya M, Wang Z, Gallagher D, St-Onge MP, Albu J, Heymsfield SB, Heshka S (1985). Total body skeletal muscle and adipose tissue volumes: estimation from a single abdominal cross-sectional image. J Appl Physiol.

[39] Chrzan R, Gleń A, Bryll A, Urbanik A. Computed tomography assessment of brain atrophy in centenarians. Int J Environ Res Public Health. 2019;16(19). 10.3390/ijerph16193659 PMC6801833 31569457

[40] Harper L, Barkhof F, Fox NC, Schott JM (2015). Using visual rating to diagnose dementia: a critical evaluation of MRI atrophy scales. J Neurol Neurosurg Psychiatry.

[41] Wahlund LO, Westman E, van Westen D, Wallin A, Shams S, Cavallin L, Larsson EM (2017). Imaging biomarkers of dementia: recommended visual rating scales with teaching cases. Insights Imaging.

[42] Meese W, Kluge W, Grumme T, Hopfenmüller W (1980). CT evaluation of the CSF spaces of healthy persons. Neuroradiology.

[43] Hu P, Uhlich R, White J, Kerby J, Bosarge P (2018). Sarcopenia measured using masseter area predicts early mortality following severe traumatic brain injury. J Neurotrauma.

[44] Deng C-Y, Lin Y-C, Wu JS, Cheung Y-C, Fan C-W, Yeh K-Y, McMahon CJ (2018). Progressive sarcopenia in patients with colorectal cancer predicts survival. AJR Am J Roentgenol.

[45] Wattjes MP, Henneman WJ, van der Flier WM, de Vries O, Träber F, Geurts JJ, Scheltens P, Vrenken H, Barkhof F (2009). Diagnostic imaging of patients in a memory clinic: comparison of MR imaging and 64-detector row CT. Radiology.

[46] Martone AM, Marzetti E, Calvani R, Picca A, Tosato M, Bernabei R, Landi F (2019). Assessment of sarcopenia: from clinical practice to research. J Gerontol Geriatr.

[47] Gu DH, Kim MY, Seo YS, Kim SG, Lee HA, Kim TH, Jung YK, Kandemir A, Kim JH, An H (2018). Clinical usefulness of psoas muscle thickness for the diagnosis of sarcopenia in patients with liver cirrhosis. Clin Mol Hepatol.

[48] Engelmann C, Schob S, Nonnenmacher I, Werlich L, Aehling N, Ullrich S, Kaiser T, Krohn S, Herber A, Sucher R (2018). Loss of paraspinal muscle mass is a gender-specific consequence of cirrhosis that predicts complications and death. Aliment Pharmacol Ther.

[49] Choe EK, Lee Y, Kang HY, Choi SH, Kim JS. Association between CT-Measured Abdominal Skeletal Muscle Mass and Pulmonary Function. J Clin Med. 2019;8(5). 10.3390/jcm8050667 PMC6572332 31083639

[50] Uhlich R, Hu P (2018). Sarcopenia diagnosed using masseter muscle area predictive of early mortality following severe traumatic brain injury. Neural Regen Res.

[51] Cruz-Jentoft AJ, Bahat G, Bauer J, Boirie Y, Bruyère O, Cederholm T, Cooper C, Landi F, Rolland Y, Sayer AA (2019). Sarcopenia: revised European consensus on definition and diagnosis. Age Ageing.

[52] Nishigori T, Obama K, Sakai Y (2020). Assessment of body composition and impact of sarcopenia and sarcopenic obesity in patients with gastric cancer. Transl Gastroenterol Hepatol.

[53] Pegueroles J, Jiménez A, Vilaplana E, Montal V, Carmona-Iragui M, Pané A, Alcolea D, Videla L, Casajoana A, Clarimón J (2018). Obesity and Alzheimer's disease, does the obesity paradox really exist? A magnetic resonance imaging study. Oncotarget.

[54] Fitzpatrick AL, Kuller LH, Lopez OL, Diehr P, O'Meara ES, Longstreth WT, Luchsinger JA (2009). Midlife and late-life obesity and the risk of dementia: cardiovascular health study. Arch Neurol.

[55] García-Ptacek S, Faxén-Irving G, Cermáková P, Eriksdotter M, Religa D (2014). Body mass index in dementia. Eur J Clin Nutr.

[56] Lee I, Cho J, Hong H, Jin Y, Kim D, Kang H (2018). Sarcopenia is associated with cognitive impairment and depression in elderly Korean women. Iran J Public Health.

[57] Walston J, Hadley EC, Ferrucci L, Guralnik JM, Newman AB, Studenski SA, Ershler WB, Harris T, Fried LP (2006). Research agenda for frailty in older adults: toward a better understanding of physiology and etiology: summary from the American Geriatrics Society/National Institute on Aging Research Conference on Frailty in Older Adults. J Am Geriatr Soc.

[58] Buford TW, Anton SD, Judge AR, Marzetti E, Wohlgemuth SE, Carter CS, Leeuwenburgh C, Pahor M, Manini TM (2010). Models of accelerated sarcopenia: critical pieces for solving the puzzle of age-related muscle atrophy. Ageing Res Rev.

[59] Erickson KI, Hillman C, Stillman CM, Ballard RM, Bloodgood B, Conroy DE, Macko R, Marquez DX, Petruzzello SJ, Powell KE (2019). Physical activity, cognition, and brain outcomes: a review of the 2018 physical activity guidelines. Med Sci Sports Exerc.

[60] Villareal DT, Holloszy JO (2006). DHEA enhances effects of weight training on muscle mass and strength in elderly women and men. Am J Physiol Endocrinol Metab.

[61] Yilmaz C, Karali K, Fodelianaki G, Gravanis A, Chavakis T, Charalampopoulos I, Alexaki VI (2019). Neurosteroids as regulators of neuroinflammation. Front Neuroendocrinol.

[62] Pan X, Wu X, Kaminga AC, Wen SW, Liu A. Dehydroepiandrosterone and Dehydroepiandrosterone Sulfate in Alzheimer's Disease: A Systematic Review and Meta-Analysis. Frontiers in Aging Neuroscience. 2019;11(61). 10.3389/fnagi.2019.00061 PMC6449476 30983988

[63] Kwon YN, Yoon SS (2017). Sarcopenia: neurological point of view. J Bone Metab.

[64] Kwan P (2013). Sarcopenia, a neurogenic syndrome?. J Aging Res.

[65] Bherer L, Erickson KI, Liu-Ambrose T (2013). Physical exercise and brain functions in older adults. J Aging Res.

[66] Perry DC, Kramer JH (2015). Reward processing in neurodegenerative disease. Neurocase.

[67] Guo X, Steen B, Matousek M, Andreasson LA, Larsson L, Palsson S, Sundh V, Skoog I (2001). A population-based study on brain atrophy and motor performance in elderly women. J Gerontol: Ser A.

[68] Singh T, Newman AB (2011). Inflammatory markers in population studies of aging. Ageing Res Rev.

[69] Rong Y-D, Bian A-L, Hu H-Y, Ma Y, Zhou X-Z (2018). Study on relationship between elderly sarcopenia and inflammatory cytokine IL-6, anti-inflammatory cytokine IL-10. BMC Geriatr.

[70] Kwon HS, Koh S-H (2020). Neuroinflammation in neurodegenerative disorders: the roles of microglia and astrocytes. Translat Neurodegener.

[71] Janowitz D, Habes M, Toledo JB, Hannemann A, Frenzel S, Terock J, Davatzikos C, Hoffmann W, Grabe HJ (2020). Inflammatory markers and imaging patterns of advanced brain aging in the general population. Brain Imaging Behav.

[72] Pasco JA, Williams LJ, Jacka FN, Stupka N, Brennan-Olsen SL, Holloway KL, Berk M (2015). Sarcopenia and the common mental disorders: a potential regulatory role of skeletal muscle on brain function?. Curr Osteoporos Rep.

