# Supplementary material for: Brain CT can predict low lean mass in the elderly with cognitive impairment: a community-dwelling study
Source: BMC Geriatr. 2022 Jan 3;22:3. doi: 10.1186/s12877-021-02626-8 (PMC8722183; doi:10.1186/s12877-021-02626-8)
Supplement: Supplementary file 1 — Additional file 1 : Supplement 1. Visual rating of global cortical atrophy. Supplement 2. Abdominal CT analysis of body composition at L3 level. [file 12877_2021_2626_MOESM1_ESM.docx]

Supplement 1: Visual rating of global cortical atrophy:

The Pasquier scale, also known as the global cortical atrophy (GCA) scale, was developed to evaluate atrophy in 13 brain regions, including frontal, parieto-occipital and temporal sulcal dilation and dilation of the ventricles. Regions are assessed separately in each hemisphere with score ranged from 0 to 3 . The final score is the sum of all scores in the 13 regions.[1, 2]

Supplement 2: Abdominal CT analysis of body composition at L3 level:

Segmentation of the skeletal muscle was performed with a combination of manual selection and attenuation segmentation using Matlab (version 8.3.0, Mathworks) at the L3 vertebral body level by Yu-Ching Lin and colleagues. A threshold range of −30 HU to +150 HU was used for skeletal muscle and -190 HU to -30 HU was for adipose tissue. The ROI for the bilateral psoas, paraspinal, and abdominal wall muscles (designated as sum of transverse abdominal, external oblique, internal oblique, and rectus abdominis muscles) and visceral and subcutaneous adipose tissue was manually drawn. Total abdominal muscle designated as sum of bilateral psoas, paraspinal, and abdominal wall muscles. For body composition analysis, normalizing measures of muscle and adipose tissue area to patient stature was typically performed. Skeletal muscle index (SMI) and adipose tissue index (ATI) was calculated for each area by dividing the cross-sectional area by the square of the patient's height in meters. The mean attenuation was measured in each skeletal muscle including total muscle and adipose tissue compartment.[3]

References

1. Harper L, Barkhof F, Fox NC, Schott JM: Using visual rating to diagnose dementia: a critical evaluation of MRI atrophy scales. *J Neurol Neurosurg Psychiatry* 2015, 86:1225-1233.

2. Wahlund LO, Westman E, van Westen D, Wallin A, Shams S, Cavallin L, Larsson EM: Imaging biomarkers of dementia: recommended visual rating scales with teaching cases. *Insights Imaging* 2017, 8:79-90.

3. Deng C-Y, Lin Y-C, Wu JS, Cheung Y-C, Fan C-W, Yeh K-Y, McMahon CJ: Progressive Sarcopenia in Patients With Colorectal Cancer Predicts Survival. *AJR American journal of roentgenology* 2018, 210:526-532.
