# Supplementary material for: Brain CT can predict low lean mass in the elderly with cognitive impairment: a community-dwelling study
Source: BMC Geriatr. 2022 Jan 3;22:3. doi: 10.1186/s12877-021-02626-8 (PMC8722183; doi:10.1186/s12877-021-02626-8)
Supplement: Supplementary file 2 — Additional file 2 : Supplemental Table 1. Pairwise comparison of ROC curves between BMI, GCA, L3SMI, MSMI, Model1 and Model 2. [file 12877_2021_2626_MOESM2_ESM.docx]

**Supplemental Table 1:** Pairwise comparison of ROC curves between BMI, GCA, L3SMI, MSMI, Model1 and Model 2

| Equality of ROC areas between BMI, GCA, L3SMI, MSMI, Model 1 and Model 2 | | | | | |
| --- | --- | --- | --- | --- | --- |
|  | SE | ^3^chi2 | ^4^df | ^5^Pr>chi2 | ^6^Bonferroni Pr>chi2 |
| ^1^Model 1 vs ^2^Model 2 | 0.026 | 0.093 | 1 | 0.761 | 1.000 |
| BMI vs Model 1 | 0.051 | 10.840 | 1 | 0.001* | 0.004* |
| GCA vs Model 1 | 0.046 | 7.845 | 1 | 0.005* | 0.020* |
| L3SMI vs Model 1 | 0.053 | 12.266 | 1 | 0.001* | 0.002* |
| MSMI vs Model 1 | 0.050 | 4.761 | 1 | 0.029* | 0.116 |
| BMI vs Model 2 | 0.051 | 9.597 | 1 | 0.002* | 0.008* |
| GCA vs Model 2 | 0.046 | 6.313 | 1 | 0.012* | 0.048* |
| L3SMI vs Model 2 | 0.053 | 6.889 | 1 | 0.009* | 0.035* |
| MSMI vs Model 2 | 0.050 | 8.483 | 1 | 0.004* | 0.014* |
| BMI vs L3SMI | 0.051 | 0.035 | 1 | 0.851 | 1.000 |
| GCA vs L3SMI | 0.046 | 0.452 | 1 | 0.501 | 1.000 |
| MSMI vs L3SMI | 0.050 | 0.605 | 1 | 0.437 | 1.000 |
| GCA vs MSMI | 0.046 | 0.001 | 1 | 0.970 | 1.000 |
| BMI vs MSMI | 0.051 | 0.368 | 1 | 0.544 | 1.000 |
| BMI vs GCA | 0.051 | 0.267 | 1 | 0.606 | 1.000 |

^1^Model 1: combination of BMI, GCA and L3SMI
^2^Model 2: combination of BMI, GCA and MSMI
^3^chi2: critical values of chi-square distributions

^4^df: degree of freedom

^5^Pr>chi2: P-value, * p < 0.05

^6^Bonferroni Pr>chi2: Bonferroni-adjusted p-value, * p < 0.05
Abbreviations: BMI, Body mass index; GCA, Global cortical atrophy; L3SMI, L3 skeletal muscle index; MSMI, Masseter skeletal muscle index; SE, Standard error
