# Supplementary material for: Brain CT can predict low lean mass in the elderly with cognitive impairment: a community-dwelling study
Source: BMC Geriatr. 2022 Jan 3;22:3. doi: 10.1186/s12877-021-02626-8 (PMC8722183; doi:10.1186/s12877-021-02626-8)
Supplement: Supplementary file 3 — Additional file 3 : Supplemental Figure 1. Participant flow charts. [file 12877_2021_2626_MOESM3_ESM.docx]

Supplemental Figure 1: Participant flow charts

1. Missing data involving abdominal and brain CT, DEXA, demographic data and CDR (n=51)
2. Age < 65 years (n=32)
3. CDR = 0 (n=24)
4. Having difficulty performing basic activities of daily living (n=33)
5. Under treatment for cancer in the prior three years (n=17)
6. Brain CT showed organic brain lesion such as hematoma, brain tumor, acute stroke, or post-infarction encephalomalacia resulting in mass effect or asymmetry affecting the ventricular system shape or subarachnoid space volume (n=32)
7. Having other neurologic or psychiatric illness and psychotropic medication usage or substance abuse (n=13)

Definition of low lean mass according to the cut-off values for ASMI by AWGS 2019 reference

Cross-sectional study during 2017 to 2019

N=81

Low ASMI

N=38

Normal ASMI

N=43

Individuals invited to participate in the study during 2017 to 2019

N=168
